# American Arctic Research

**Published:** 1881-04

**Authors:** 


### AMERICAN ARCTIC RESEARCH.

One of the items of the Sundry Civil Appropriation Bill of the late Congress was $175.000 for an expedition to the regions north of Behring's Straight for relief of the Jeannette and the missing whalers. The steam whaler Mary and Helen, of New Bedford, now at San Francisco, has been purchased for the purpose. The price paid was $100.000, thus leaving $75.000 to be used in making the vessel ready for the new service and providing for her outfit

It is announced that two other expeditions, under the direction of General Hazen, Chief Signal Officer, will be dispatched to the north next summer for purely scientific investigations. One of these to be commanded by Lieutenant Greely, of the Signal Corps, will go to Lady Franklin Bay; the other to the north coast of Alaska. The Washington correspondent of the *Tribune* says that Professor Baird of the Smithsonian Institution, and Captain Patterson, of the Coast Survey, are co-operating with General Hazen and will each be represented in one or both of these expeditions. These enterprises are a part of polar observation in which several European nations are participants with this country. Russia has promised to occupy two stations, one at the mouth of the Lena in Eastern Siberia and the other on the New Siberian Island, which is some distance east of Wrangel Land. Sweden has promised to occupy North Cape in Finland. Denmark will establish a station at Upernavik, Greenland. Germany

—though she has not made an absolute promise to do so—is expected to send an expedition to the island of Jan Mayen, east of Greenland. Holland will occupy the mouth of the Ob and Spitzbergen. Austria, represented by Count Wilszek and Lieutenant Weyprecht, will occupy Nova Zembla. Canada will probably occupy Melville Island. Italy will fit out an expedition to the Southern Hemisphere, and will probably select its location on Cape Horn. It is also expected that the Island of Georgia, in the Southern Hemisphere, will be occupied by an expedition from some other European nation.

Lieutenant Greeley's expedition will consist of three officers of the army and twenty-one enlisted men. It will be assembled in Washington, not later than the 15th of May, and in St. John's, Newfoundland, about one month later. It is expected that the expedition will leave St. John's about the 1st of July, and touching at Disco, will take on board Dr. Pavey, the naturalist of the expedition, who has been in Greenland during the last winter collecting dogs, sledges and other material for the expedition. Several teams of dogs will be taken from Disco and Upernavik with two Esquimaux hunters. The vessel is expected to reach Lady Franklin Bay by the last of August, when, disembarking the party, it will return to the United States.

In addition to the scientific observations to be made by the permanent party, the northern coast of Greenland probably will be explored, and it is believed that the question as to whether Greenland is an island or a continent can be settled, and also whether land exists to the northward of Cape Britannia, the furtherest point seen by the English expedition of 1865. The station is to be visited annually by vessels which will bring fresh supplies and a number of new recruits, in order that those unfitted for the work by reason of disease or otherwise may return to the United States. Lieutenant F. E. Kislingbury, of the 11th infantry, an officer who has already made a creditable record as a scientific man has been designated as the geographer of the expedition. The third officer of the expedition has not yet been selected.

The meteorological observations of the expedition will be made by a party of four Signal Service observers specially trained for it. Mr. William Rice, of Washington, who will probably be the photographer of the expedition, has already had experience in photography within the Artic circle. The enlisted force is to be selected from a large number of volunteers who have seen difficult service in the extreme Northwest, and who for that reason are better prepared to resist the rigors of the Arctic winter. Lieutenant Greeley's experience as a Signal officer will, it is expected, be found of great value in making communications from point to point. For this purpose he will use the Myers signal code, sending signals by the heliograph for distances of forty miles when the sun shines, and by flags and lanterns for shorter distances at other times. This will be an advantage which former expeditions have not possessed.—*Scientific American.*

